# Osteomyelitis of a long bone due to *Fusobacterium nucleatum* and *Actinomyces meyeri* in an immunocompetent adult: A case report and literature review

**DOI:** 10.1186/1471-2334-12-161

**Published:** 2012-07-20

**Authors:** Min Ji Lee, Young Eun Ha, Hye Yon Park, Jun Hee Lee, Yoon Jung Lee, Ki Sun Sung, Cheol-In Kang, Doo Ryeon Chung, Jae-Hoon Song, Kyong Ran Peck

**Affiliations:** 1 Department of Internal Medicine, Samsung Medical Center, Sungkyunkwan University School of Medicine, Seoul, Republic of Korea; 2 Department of Orthopedic Surgery, Samsung Medical Center, Sungkyunkwan University School of Medicine, Seoul, Republic of Korea

**Keywords:** *Fusobacterium nucleatum*, Osteomyelitis, Periodontitis

## Abstract

**Background:**

*Fusobacterium* species are uncommon causes of osteomyelitis. These organisms are normal flora of the oral cavity. Therefore, they mostly cause osteomyelitis of the head and neck. Hematogenous osteomyelitis at distant sites other than the head and neck has rarely been reported in pediatric or immunocompromised patients. Here, we report the first case of osteomyelitis of a long bone combined with a muscle abscess due to *Fusobacterium nucleatum* in an otherwise healthy adult.

**Case presentation:**

A 59-year-old Korean man was admitted for pain and swelling of the right lower leg, which had been persistent for two weeks. Magnetic resonance imaging showed osteomyelitis of the right fibula with a surrounding muscle abscess of the right lower leg. Incision and drainage was performed, and repetitive tissue cultures grew *F. nucleatum.* In this patient, it was presumed that recurrent periodontitis caused hematogenous seeding of *F. nucleatum* to a distant site leading to osteomyelitis with a muscle abscess. The patient was successfully treated with intravenous ampicillin-sulbactam for three weeks and oral amoxicillin-clavulanate for eight weeks. He also underwent repeated surgical drainage. He has no evidence of recurrence after seven months of follow-up.

**Conclusions:**

Clinicians should be aware that *F. nucleatum* could be the etiologic agent of hematogenous osteomyelitis of a long bone in an immunocompetent patient.

## Background

*Fusobacterium* species are gram-negative bacilli that are nonmotile, non-sporulating, obligate anaerobes from the family Bacteroidaceae [1]. They have frequently been isolated from a wide variety of clinically significant anaerobic infections, including oral and dental infections, brain abscesses, bacteremia, endocarditis, and soft tissue infections [2-5]. Only occasionally have *Fusobacterium* species been isolated from bone and joint infections. They are part of the normal flora of the oral cavity, gastrointestinal tract, upper respiratory tract, and female genital tract [2]. They are mostly found in the mouth and are discovered to a lesser extent in feces and the urogenital tract [3]. Therefore, reported cases of osteomyelitis caused by *Fusobacterium* spp. were mostly in the head and neck area. These were associated with chronic periodontitis or an odontogenic abscess and resulted from contiguous spread of the infection. For cases of osteomyelitis resulting from hematogenous seeding, most patients were children or had predisposing factors that could easily lead to osteomyelitis. Such risk factors include indwelling intravascular catheters, distant foci of infection, intravenous drug abuse, vascular insufficiency, sickle cell disease, traumatic bone injury, open fractures, or chronic soft tissue infections [1,4-8]. Our literature review revealed that there are no reports of long bone osteomyelitis caused by *Fusobacterium* spp. in immunocompetent adults. Here we describe a case of fibular osteomyelitis combined with muscle abscess caused by *F. nucleatum* in an adult patient with no known predisposing factors.

## Case presentation

A 59-year-old previously healthy man presented with fever and pain and swelling in his right lower leg. About two months before this admission, he had developed pain in his right lower leg. The pain had progressively worsened despite analgesics, and his right lower leg had begun to swell. About two weeks before this admission, he had been admitted to an outside hospital where he was found to have osteomyelitis of the right fibula combined with abscesses of adjacent muscles (soleus, tibialis posterior, and fibularis longus). He underwent incision and drainage of his right leg. A first generation cephalosporin was started empirically. However, repeated cultures from tissues had grown no microorganisms, and the leg had been draining pus persistently up until transfer to our hospital. The patient was transferred to our hospital for further diagnostic evaluation and treatment.

His past medical history was negative for diabetes mellitus, arterial hypertension, alcoholism, steroid use, and any other systemic infections. He has been smoking one packet of cigarettes a day for 15 years. He denied a history of local trauma or recreational drug abuse. He recalled recurrent periodontitis lasting for ten years. Approximately three months before presentation, he had four teeth extracted and dentures implanted. He did not receive prophylactic antibiotics before the tooth extraction.

**Figure 1:**
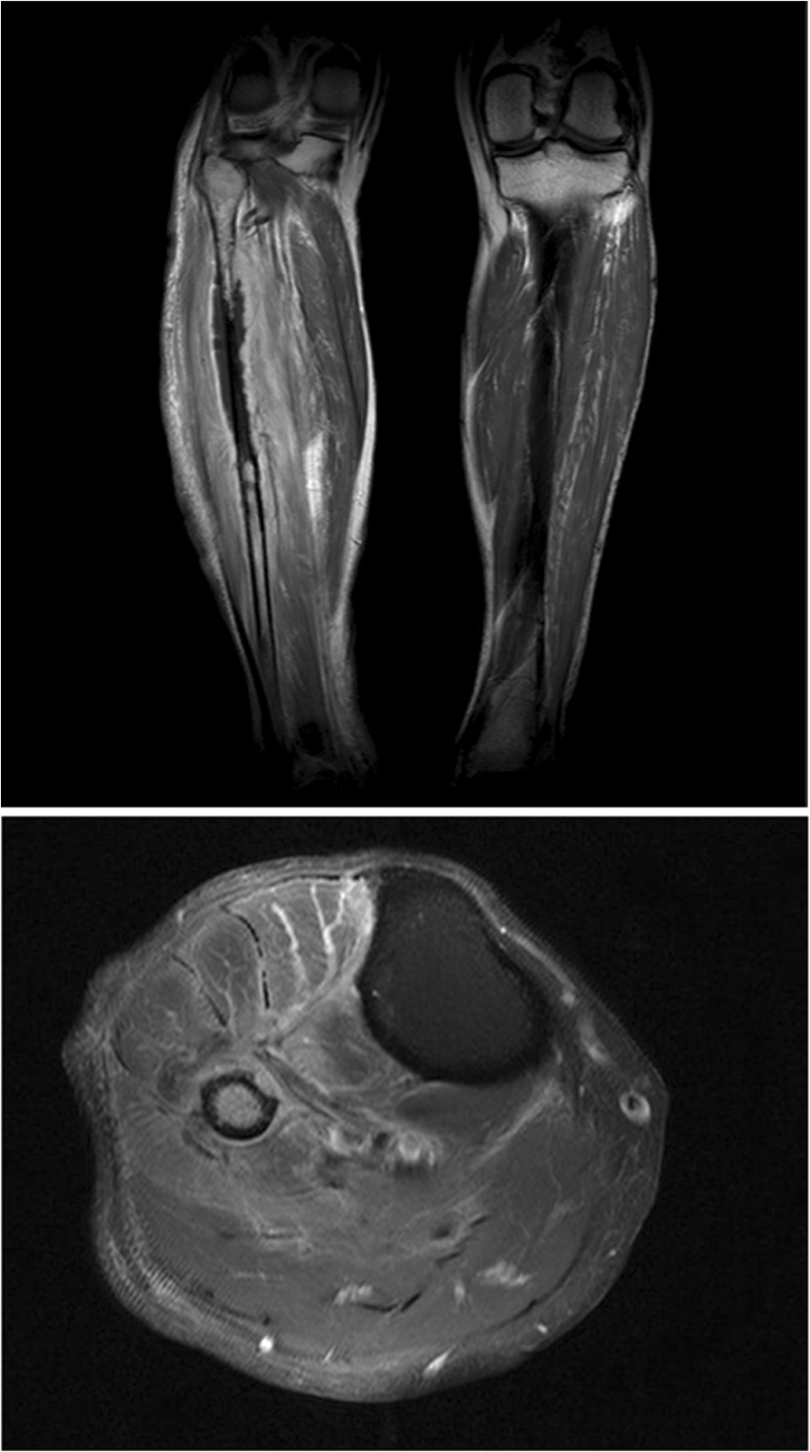
**Enhanced magnetic resonance imaging of the right lower leg showing fibular enhancement and adjacent muscular abscess.** (Fat saturated contrast enhanced T1W1).

On physical examination, temperature was 37.1 °C, blood pressure was 124/69 mmHg, pulse was 100 beats per minute, and respiratory rate was 18 breaths per minute. In general, he appeared ill, although his mental status was alert and oriented. The wound on the lateral side of his right lower leg had an incision 16 cm in size. The fibula was exposed with signs of inflammation of the adjacent muscles with draining pus and a foul odor. Laboratory evaluation revealed a leukocyte count of 12,980/μL (80% neutrophils), a hemoglobin of 9.7 g/dL, a platelet count of 385,000/μL, a C-reactive protein level of 329 mg/L, and an erythrocyte sedimentation rate of 86 mm/h. A chest radiograph demonstrated no active lung lesions. Computed tomography (CT) of the lower extremities at the outside hospital revealed osteomyelitis of the right fibula and a muscular abscess along the fibular shaft. Magnetic resonance imaging of the lower extremities (Figure 1), which was performed at our hospital, revealed a slightly decreased amount of surrounding abscess. MRI also showed persistent osteomyelitis of the right fibula with no change in the periostitis of the right proximal tibia compared to CT imaging from the outside hospital.

Incision and drainage were performed at the bedside and tissues were sent for Gram staining and culture. Intravenous ampicillin-sulbactam was started at 3 g every six hours. On hospital day 3, fibular excision was performed due to the severe osteomyelitis. Findings on microscopic examination of the bone biopsy specimen were consistent with acute osteomyelitis and showed necrotic tissue with microabscess. Initial tissue culture at our hospital revealed *F. nucleatum* and *Actinomyces meyeri* as the causative organisms. Tissue cultures performed at the outside hospital also reportedly grew *Fusobacterium* spp*.* in two out of five specimens. The patient had repeat incision and debridement surgery on hospital day 7 and day 17. Intravenous ampicillin-sulbactam was administered for three weeks and then switched to oral amoxicillin-clavulanate at 625 mg every 8 h for eight weeks. Antibiotics were discontinued after 11 weeks of successful treatment. His recovery has been uneventful without recurrence of infection after seven months of follow-up.

## Discussion

Anaerobes have traditionally been viewed as uncommon causes of osteomyelitis because they are difficult to isolate from infectious sites due to their fastidious nature. However, due to the development of methods for detecting anaerobes, reports of anaerobic osteomyelitis have been increasing; up to 39% of cases of osteomyelitis were associated with anaerobic infections in a previous study [9]. Anaerobic osteomyelitis has typically been reported in patients with complicated bone fractures or underlying chronic disease, usually as a result of non-hematogenous spread. The predominant anaerobes causing osteomyelitis are *Bacteroides* spp*., Peptostreptococcus* spp*., Fusobacterium* spp*., Clostridium* spp*.* and *Propionibacterium acnes*[9,10].

*Fusobacterium* spp*.* are commonly found in periodontal disease, and they are known to produce tissue irritants such as butyric acid, proteases, and cytokines. They have strong adhesive properties due to the presence of lectins. These outer membrane proteins mediate adhesion to epithelia and tooth surfaces and coagglutination with other suspected pathogens [3].

*Fusobacterium* spp*.,* which are members of the normal oral flora, are most frequently isolated from cranial or facial infections [10]. The species most commonly isolated is *F. necrophorum*[6,7,11]. Cases of *F. nucleatum* are relatively rare [1,9]. Table 1 shows cases of *Fusobacterium* osteomyelitis at sites other than the head and neck that have been reported in the medical literature. As indicated, most of the cases are children or patients with predisposing factors for anaerobic hematogenous osteomyelitis. To our knowledge, our patient is the first case of long bone osteomyelitis caused by *F. nucleatum* in an immunocompetent adult. Many patients with anaerobic osteomyelitis have an anaerobic infection elsewhere in the body that is the source of the organisms involved in osteomyelitis. Osteomyelitis of long bones is generally due to hematogenous spread, trauma, or the presence of a prosthetic device [10]. Our patient had no specific infection source except a history of recurrent periodontitis. Patients with periodontal disease are predisposed to systemic infections with anaerobic bacteria such as *Fusobacterium* spp. There are a few case reports of long bone osteomyelitis following oral infection [12]. In our patient, his poor dentition may have caused *F. nucleatum* bacteremia, leading to hematogenous osteomyelitis of the lower leg with an abscess of the adjacent muscle.

**Table 1:** Cases of osteomyelitis caused by *Fusobacterium* spp. reported in the medical literature

**Cases**	**Gender/Age (year)**	**Predisposing factors**	**Location**	**Species**	**Culture specimens**	**Antibiotics**	**Outcome**
Murray *et al.*[4]	M/7	Sickle cell disease	Tibia	*F.nucleatum*	Tissue	Nafcillin + Ceftriaxone → Clindamycin 6w (IV)	Cured
Beauchamp *et al.*[5]	M/6	None	Pelvis	*F.nucleatum*	Tissue	Clindamycin 10 w (IV 4w, PO 6w)	Cured
Le Moal *et al.*[6]	F/78	Recent dental work	Vertebra, L5-S1	*F.necrophorum*	Blood and tissue	Clindamycin 12 w (IV 4w, PO 8w)	Cured
	M/62	Arterial hypertension	Vertebra, L4-L5	*F.necrophorum*	Blood	Amoxicillin-clavulanic acid → Clindamycin 8w (IV 4w, PO 4w)	Cured
	M/61	Diabetes mellitus, Arterial hypertension	Vertebra, T6-T8	*F.nucleatum*	Blood and tissue	Penicilin G 4w (IV) → Clindamycin 8w (PO)	Cured
Sanchez *et al.*[1]	M/16	None	Pubic symphysis	*F.necrophorum*	Blood	Imipenem → Amoxicillin-clavulanic acid 6w	Cured
Stahlman *et al.*[7]	M/13	None	Fibula	*F.necrophorum*	Tissue	Penicilin G 6w (IV)	Cured

Management of osteomyelitis includes symptomatic therapy, immobilization for some patients, adequate drainage of purulent material, and antibiotic therapy consisting of parenteral administration of antibiotics for at least four to eight weeks. In some cases, even longer antibiotic treatment is necessary [13]. *Fusobacterium* spp*.* are commonly sensitive to the usual anti-anaerobic antibacterial agents including penicillin G, clindamycin, metronidazole, chloramphenicol, imipenem, and cefoxitin [7]. However, there is evidence of emerging resistance of some *Fusobacterium* spp*.* isolates to penicillins, carbapenems, and clindamycin [13,15]. The first description of a β-lactamase in *Fusobacterium* spp*.* was reported in 1985, and this was shown to primarily be a penicillinase with little activity against cephalosporins [15]. Since then, several studies have reported β-lactamase production by *Fusobacterium* spp*.* Fatal sepsis due to a β-lactamase-producing strain occurred in an immunocompromised patient [16]. In studies done from 1999 to 2008, 0–14.6% of *Fusobacterium* spp*.* isolates were found to be nonsusceptible to penicillin [17,24]. The nonsusceptibility to β-lactam-β-lactamase inhibitors was found to be much lower (0-3.3%) [18-22]. There were regional differences between species in terms of penicillin resistance, but resistance to β-lactam-β-lactamase inhibitors was similarly low in different regions [24]. Recently, as *Bacteroides* spp*.* have become more resistant to carbapenem, carbapenem resistance in *Fusobacterium* spp. has also been reported. In a study from Taiwan, 4% of *Fusobacteria* isolates were “nonsusceptible” to imipenem and 8% were “nonsusceptible” to meropenem [17]. Our patient was successfully treated with β-lactam-β-lactamase inhibitors. The duration of antibiotic therapy is debated, but prolonged duration of high-dose β-lactam therapy is recommended because of the endovascular nature of the infection. Surgical debridement is crucial given the tendency toward abscess formation [7].

## Conclusions

We reported the first case of osteomyelitis caused by *F. nucleatum* in an adult patient with no definite risk factors for hematogenous osteomyelitis. It is believed that in this patient, recurrent periodontitis might have been the source of bacteremia. Efforts to isolate anaerobic pathogens should be made in patients with characteristics of anaerobic infections, such as large abscesses.

## Consent

Written informed consent was obtained from the patient for publication of this case report and any accompanying images. A copy of the written consent is available for review by the Series Editor of this journal.

## Competing interests

The authors declare that they have no competing interests.

## Authors’ contributions

MJ Lee managed the patient and drafted and revised the manuscript. YE Ha reviewed the manuscript. YJ Lee, HY Park, and JH Lee were involved in the patient’s clinical care and contributed to the drafting of the manuscript. KS Sung provided the surgical support in the patient’s clinical care. CI Kang, DR Chung, JH Song, and KR Peck contributed to coordinating the manuscript submission and drafting. All authors have read the manuscript and approved its final version.

## Pre-publication history

The pre-publication history for this paper can be accessed here:

http://www.biomedcentral.com/1471-2334/12/161/prepub
